# 9-*O*-Ethyl­berberrubinium iodide monohydrate

**DOI:** 10.1107/S1600536810036664

**Published:** 2010-09-18

**Authors:** Peter Grundt, Jennifer Pernat, Bogdana Krivogorsky, Melanie A. Halverson, Steven M. Berry

**Affiliations:** a Department of Chemistry and Biochemistry, University of Minnesota Duluth, 1039 University Dr., Duluth, MN 55812, USA

## Abstract

In the title compound (systematic name: 9-eth­oxy-10-meth­oxy-5,6-dihydro-1,3-dioxolo[4,5-*g*]isoquinolino­[3,2-*a*]isoquin­olin-7-ium iodide monohydrate), 2C_21_H_20_NO_4_^+^·2I^−^·H_2_O, two independent mol­ecules pack in the unit cell, where interactions between the molecules are stabilized by weak inter­molecular π–π stacking inter­actions [centroid–centroid distances in the range 3.571 (4) to 3.815 (4)Å]. Inter­molecular C—H⋯O inter­actions are also observed. The iodide anions are disordered with occupancy ratios of 0.94 (1):0.06 (1) and 0.91 (1):0.09 (1). The cationic molecule is planar in structure with a small torsion resulting from the dihydropyridine ring.

## Related literature

For the synthesis of the title compound, see: Iwasa *et al.* (1997 ▶). The title compound is a derivative of the natural product berberine. For the anti-parasitic activity of berberine and its derivatives, see: Nkwengoua *et al.* (2009 ▶); Acero *et al.* (1995 ▶); Ghosh *et al.* (1985 ▶); Wright *et al.* (2000 ▶); Iwasa *et al.* (1998 ▶); Sheng *et al.* 1997 ▶); McCall *et al.* (1994 ▶). For a related structure, see: Chen *et al.* (2009 ▶). For the Chebychev weighting scheme, see: Prince (1982 ▶); Watkin (1994 ▶). 
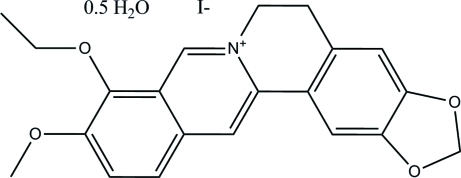


## Experimental

### 

#### Crystal data

2C_21_H_20_NO_4_^+^·2I^−^·H_2_O
*M* *_r_* = 972.61
Triclinic, 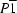

*a* = 11.036 (3) Å
*b* = 13.449 (4) Å
*c* = 14.086 (4) Å
α = 80.442 (6)°
β = 71.121 (5)°
γ = 76.875 (5)°
*V* = 1916.8 (9) Å^3^
*Z* = 2
Mo *K*α radiation
μ = 1.70 mm^−1^
*T* = 93 K
1.00 × 0.10 × 0.10 mm

#### Data collection

Rigaku R-AXIS RAPID II image plate diffractometer
Absorption correction: multi-scan (*ABSCOR*; Higashi, 1995 ▶) *T*_min_ = 0.469, *T*_max_ = 0.712
50086 measured reflections
8751 independent reflections
4563 reflections with *I* > 2.0σ(*I*)
*R*_int_ = 0.101

#### Refinement

*R*[*F* ^2^ > 2σ(*F* ^2^)] = 0.059
*wR*(*F* ^2^) = 0.195
*S* = 0.96
8751 reflections
502 parameters
12 restraints
All H-atom parameters refined
Δρ_max_ = 1.75 e Å^−3^
Δρ_min_ = −2.26 e Å^−3^

### 

Data collection: *CrystalClear* (Rigaku, 2009 ▶); cell refinement: *HKL-2000* (Otwinowski & Minor, 1997 ▶); data reduction: *CrystalClear*; program(s) used to solve structure: *CrystalStructure* (Rigaku, 2009 ▶) and *SIR2004* (Burla *et al.*, 2005 ▶); program(s) used to refine structure: *CRYSTALS* (Betteridge *et al.*, 2003 ▶); molecular graphics: *CAMERON* (Watkin *et al.*, 1996 ▶); software used to prepare material for publication: *CRYSTALS*.

## Supplementary Material

Crystal structure: contains datablocks global, I. DOI: 10.1107/S1600536810036664/jj2056sup1.cif

Structure factors: contains datablocks I. DOI: 10.1107/S1600536810036664/jj2056Isup2.hkl

Additional supplementary materials:  crystallographic information; 3D view; checkCIF report

## Figures and Tables

**Table 1:** Hydrogen-bond geometry (Å, °)

*D*—H⋯*A*	*D*—H	H⋯*A*	*D*⋯*A*	*D*—H⋯*A*
C9*A*—H10*A*⋯O5	0.93	2.47	3.358 (11)	159
C21*B*—H20*B*⋯O1*B*^i^	0.96	2.51	3.466 (11)	177

Symmetry code: (i) 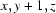.

**Fig. 1.**
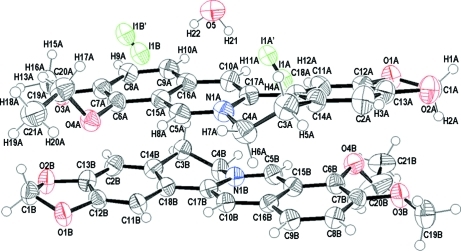
The title compound with displacement ellipsoids drawn at the 50% probability level. H atoms are shown as spheres of arbitrary radius. H atom labels are omitted from molecule B for clarity.

**Fig. 2.**
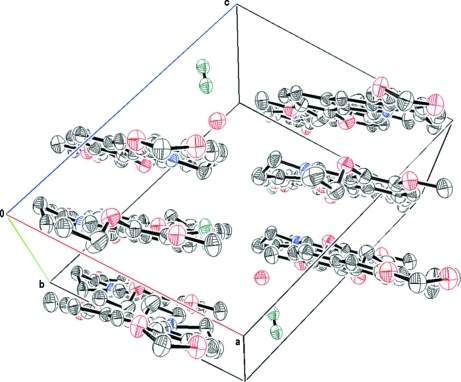
The packing diagram of the title compound with displacement ellipsoids drawn at the 50% probability level. Hydrogen atoms are omitted for clarity.

## supplementary crystallographic information

### Comment

The title compound, a derivative of the natural product berberine, is of interest with respect to its anti-parasite activity and biological properties Chen *et al.* (2009). Of particular interest is the discovery that berberine and its analogs inhibit the growth of strains of the parasites Leishmania (Nkwengoua *et al.*, 2009, Acero *et al.*, 1995, Ghosh *et al.*, 1985), Trypanosoma (Nkwengoua *et al.*, 2009), and Plasmodium (Wright *et al.*, 2000, Iwasa *et al.*, 1998, Sheng *et al.*, 1997, McCall *et al.*, 1994). Based on these findings and in our continued effort to characterize compounds that may inhibit the life cycle of the parasite Toxoplasma gondii we have synthesized the 9-ethyl analog of berberine.

The title compound, 2(C_21_H_20_NO_4_^+^), 2(I^-^), H_2_O, crystallizes in the P-1 space group with two independent molecules in the unit cell. A solvent water molecule occupies the lattice at H-bonding distance from the disordered iodide (1.94 & 0.06) anions [O5—I1A 3.509 (6) and O5—I1B 3.600 (6) Å]. The compound crystallizes with a slightly twisted planar structure due to the dihydropyridine moiety, which results in torsion angles of 17.7 (5)° (C15A/C16A/C11A/C2A) and 6.1 (5)° (C15B/C16B/C11B/C2B) between the planes of the isoquiniline and benzodioxole moieties for molecules A and B respectively. The molecules are layered in the crystal lattice with normal π—π stacking distances between centroids of the rings of molecules A and B of 3.571 (4)Å (rings C6A/C7A/C8A/C9A/C15A/C16A to C2B/C11B/C12B/C13B/C14B/C18B), 3.839 (4)Å (rings N1A/C5A/C10A/C15A/C16A/C17A to N1B/C5B/C10B/C15B/C16B/C17B), and 3.686 (4)Å (rings C2A/C11A/C12A/C13A/C14A/C18A to C6B/C7B/C8B/C9B/C15B/C16B). Identical molecules neighboring each other are located at further stacking distances with the closest controid distance of 3.815 (4)Å between molecules A to A and 3.949 (4)Å between molecules B to B.

### Experimental

The title compound was prepared by vacuum pyrolysis of berberine hydrochloride followed by alkylation with ethyl iodide (Iwasa *et al.*, 1997). The crystals suitable for single-crystal X-ray diffraction were grown in DMSO-d6. The crystal was diffracted in the cold stream of an X-Stream 2000 Liquid nitrogen generator with an open-flow nitrogen cryostat with a nominal stability of 0.1 K.

### Refinement

The H atoms were all located in a difference map, but those attached to carbon atoms were repositioned geometrically. The H atoms were initially refined with soft restraints on the bond lengths and angles to regularize their geometry (C—H in the range 0.93–0.98, O—H = 0.82 Å) and *U*_iso_(H) (in the range 1.2–1.5 times *U*_eq_ of the parent atom), after which the positions were refined with riding constraints. The ethyl groups on the molecules were found to have some disorder, with the methylene carbon (C20B) demonstrating the largest thermal parameters. The disordered iodide anions were solved with two partially occupied positions. The anisotropic parameters U_xy_ were restrained for each of the iodide pairs during refinement.

### Crystal data

**Table:**

2C_21_H_20_NO_4_^+^·2I^−^·H_2_O	*Z* = 2
*M**_r_* = 972.61	*F*(000) = 972
Triclinic, *P*1	*D*_x_ = 1.685 Mg m^−^^3^
Hall symbol: -P 1	Melting point: 257 K
*a* = 11.036 (3) Å	Mo *K*α radiation, λ = 0.71073 Å
*b* = 13.449 (4) Å	Cell parameters from 50086 reflections
*c* = 14.086 (4) Å	θ = 3–27.5°
α = 80.442 (6)°	µ = 1.70 mm^−^^1^
β = 71.121 (5)°	*T* = 93 K
γ = 76.875 (5)°	Needle, yellow
*V* = 1916.8 (9) Å^3^	1.00 × 0.10 × 0.10 mm

### Data collection

**Table:**

Rigaku R-AXIS RAPID II image plate diffractometer	8751 independent reflections
Radiation source: Mo sealed tube	4563 reflections with *I* > 2.0σ(*I*)
graphite	*R*_int_ = 0.101
Detector resolution: 10 pixels mm^-1^	θ_max_ = 27.5°, θ_min_ = 3.1°
ω scans	*h* = −14→14
Absorption correction: multi-scan (*ABSCOR*; Higashi, 1995)	*k* = −17→17
*T*_min_ = 0.469, *T*_max_ = 0.712	*l* = −18→18
50086 measured reflections	

### Refinement

**Table:**

Refinement on *F*^2^	Primary atom site location: structure-invariant direct methods
Least-squares matrix: full	Secondary atom site location: difference Fourier map
*R*[*F*^2^ > 2σ(*F*^2^)] = 0.059	Hydrogen site location: inferred from neighbouring sites
*wR*(*F*^2^) = 0.195	All H-atom parameters refined
*S* = 0.96	Method, part 1, Chebychev polynomial, (Watkin, 1994; *P*rince, 1982) [*w*eight] = 1.0/[A_0_*T_0_(x) + A_1_*T_1_(x) ··· + A_n-1_]*T_n-1_(x)] where A_i_ are the Chebychev coefficients listed belo*w* and x = *F* /*F*max Method = Robust Weighting (*P*rince, 1982) W = [*w*eight] * [1-(delta*F*/6*sigma*F*)^2^]^2^ A_i_ are: 4.85 6.23 1.54
8751 reflections	(Δ/σ)_max_ = 0.001
502 parameters	Δρ_max_ = 1.75 e Å^−^^3^
12 restraints	Δρ_min_ = −2.26 e Å^−^^3^
0 constraints	

### Fractional atomic coordinates and isotropic or equivalent isotropic displacement parameters (Å^2^)

**Table:**

	*x*	*y*	*z*	*U*_iso_*/*U*_eq_	Occ. (<1)
O1A	0.7061 (5)	0.4522 (4)	0.7015 (4)	0.0527	
C1A	0.5927 (8)	0.5331 (6)	0.7096 (6)	0.0517	
O2A	0.5226 (6)	0.5086 (4)	0.6480 (4)	0.0577	
C2A	0.5222 (8)	0.3441 (6)	0.5954 (6)	0.0554	
C3A	0.5468 (8)	0.1682 (6)	0.5415 (6)	0.0552	
C4A	0.5678 (7)	0.0617 (6)	0.5900 (6)	0.0511	
N1A	0.7020 (5)	0.0278 (4)	0.5983 (4)	0.0413	
C5A	0.7560 (7)	−0.0722 (5)	0.5920 (5)	0.0413	
C6A	0.9343 (6)	−0.2175 (5)	0.6016 (5)	0.0401	
C7A	1.0547 (7)	−0.2531 (5)	0.6186 (5)	0.0436	
C8A	1.1203 (6)	−0.1848 (5)	0.6375 (5)	0.0417	
C9A	1.0685 (7)	−0.0817 (5)	0.6410 (5)	0.0440	
C10A	0.8835 (6)	0.0610 (5)	0.6336 (5)	0.0398	
C11A	0.7418 (7)	0.2722 (5)	0.6670 (5)	0.0462	
C12A	0.6771 (7)	0.3709 (5)	0.6694 (5)	0.0437	
C13A	0.5686 (7)	0.4057 (6)	0.6358 (6)	0.0490	
C14A	0.5878 (7)	0.2405 (5)	0.5902 (5)	0.0438	
C15A	0.8781 (6)	−0.1118 (5)	0.6061 (4)	0.0392	
C16A	0.9456 (6)	−0.0429 (5)	0.6270 (5)	0.0411	
C17A	0.7628 (6)	0.0966 (5)	0.6204 (4)	0.0378	
C18A	0.6934 (6)	0.2049 (5)	0.6268 (5)	0.0406	
O3A	1.1012 (5)	−0.3557 (4)	0.6163 (4)	0.0471	
O4A	0.8648 (5)	−0.2821 (3)	0.5852 (3)	0.0428	
C19A	1.2226 (8)	−0.3942 (5)	0.6361 (6)	0.0497	
C20A	0.9150 (8)	−0.3152 (6)	0.4853 (5)	0.0531	
C21A	0.8288 (9)	−0.3797 (7)	0.4743 (7)	0.0662	
O1B	0.7780 (5)	−0.4304 (3)	0.8199 (4)	0.0481	
C1B	0.8993 (7)	−0.5023 (5)	0.7914 (6)	0.0507	
O2B	0.9851 (5)	−0.4741 (4)	0.8366 (4)	0.0528	
C2B	0.9914 (7)	−0.3031 (6)	0.8764 (5)	0.0471	
C3B	0.9793 (7)	−0.1200 (5)	0.9045 (5)	0.0463	
C4B	0.8775 (7)	−0.0374 (5)	0.9633 (6)	0.0468	
N1B	0.7687 (5)	−0.0013 (4)	0.9196 (4)	0.0390	
C5B	0.7139 (7)	0.0966 (5)	0.9225 (5)	0.0442	
C6B	0.5421 (7)	0.2398 (5)	0.8997 (5)	0.0433	
C7B	0.4229 (6)	0.2745 (5)	0.8790 (5)	0.0423	
C8B	0.3624 (6)	0.2055 (5)	0.8563 (5)	0.0412	
C9B	0.4177 (6)	0.1029 (5)	0.8509 (5)	0.0399	
C10B	0.6057 (6)	−0.0366 (5)	0.8629 (4)	0.0368	
C11B	0.7373 (6)	−0.2504 (5)	0.8535 (5)	0.0415	
C12B	0.8119 (6)	−0.3468 (5)	0.8398 (5)	0.0407	
C13B	0.9343 (7)	−0.3723 (5)	0.8522 (5)	0.0449	
C14B	0.9186 (6)	−0.2035 (5)	0.8885 (5)	0.0433	
C15B	0.5975 (6)	0.1356 (5)	0.8969 (5)	0.0399	
C16B	0.5391 (6)	0.0654 (5)	0.8706 (4)	0.0363	
C17B	0.7212 (6)	−0.0725 (5)	0.8861 (4)	0.0371	
C18B	0.7938 (6)	−0.1769 (5)	0.8779 (5)	0.0400	
O4B	0.6121 (5)	0.3048 (4)	0.9131 (4)	0.0498	
O3B	0.3732 (5)	0.3765 (4)	0.8823 (4)	0.0489	
C19B	0.2472 (8)	0.4115 (6)	0.8666 (7)	0.0569	
C20B	0.5681 (9)	0.3468 (11)	1.0051 (8)	0.0893	
C21B	0.6740 (9)	0.3896 (8)	1.0199 (6)	0.0634	
O5	1.2817 (6)	0.0700 (5)	0.6183 (4)	0.0635	
I1A	1.02838 (5)	0.19860 (3)	0.80552 (4)	0.0447	0.9379
I1A'	1.1037 (7)	0.1783 (5)	0.7437 (6)	0.0447	0.0621
I1B	1.39483 (6)	−0.16491 (4)	0.75430 (5)	0.0520	0.9057
I1B'	1.4616 (6)	−0.1882 (4)	0.7030 (5)	0.0520	0.0943
H1A	0.6187	0.5993	0.6853	0.0619*	
H2A	0.5380	0.5327	0.7790	0.0618*	
H3A	0.4499	0.3682	0.5718	0.0661*	
H4A	0.5966	0.1695	0.4707	0.0661*	
H5A	0.4542	0.1889	0.5483	0.0663*	
H6A	0.5042	0.0590	0.6571	0.0612*	
H7A	0.5560	0.0157	0.5490	0.0607*	
H8A	0.7107	−0.1157	0.5775	0.0495*	
H9A	1.2018	−0.2098	0.6474	0.0498*	
H10A	1.1145	−0.0368	0.6524	0.0527*	
H11A	0.9261	0.1064	0.6479	0.0480*	
H12A	0.8143	0.2501	0.6909	0.0558*	
H13A	1.2430	−0.4679	0.6357	0.0750*	
H14A	1.2168	−0.3759	0.7007	0.0748*	
H15A	1.2896	−0.3646	0.5852	0.0749*	
H16A	1.0021	−0.3568	0.4784	0.0636*	
H17A	0.9200	−0.2562	0.4342	0.0642*	
H18A	0.8548	−0.3958	0.4057	0.0992*	
H19A	0.8354	−0.4422	0.5184	0.0988*	
H20A	0.7397	−0.3432	0.4928	0.0989*	
H1B	0.8849	−0.5716	0.8167	0.0610*	
H2B	0.9354	−0.4972	0.7183	0.0609*	
H3B	1.0747	−0.3211	0.8837	0.0571*	
H4B	1.0408	−0.1494	0.9427	0.0559*	
H5B	1.0244	−0.0878	0.8384	0.0558*	
H6B	0.8441	−0.0668	1.0323	0.0558*	
H7B	0.9172	0.0200	0.9622	0.0559*	
H8B	0.7535	0.1410	0.9415	0.0528*	
H9B	0.2816	0.2301	0.8448	0.0488*	
H10B	0.3754	0.0586	0.8342	0.0478*	
H11B	0.5706	−0.0817	0.8411	0.0439*	
H12B	0.6540	−0.2340	0.8465	0.0500*	
H13B	0.2236	0.4843	0.8700	0.0849*	
H14B	0.1834	0.3776	0.9178	0.0848*	
H15B	0.2506	0.3966	0.8011	0.0848*	
H16B	0.5428	0.2942	1.0590	0.1070*	
H17B	0.4937	0.4011	1.0052	0.1071*	
H18B	0.6422	0.4194	1.0829	0.0951*	
H19B	0.7468	0.3356	1.0206	0.0951*	
H20B	0.6997	0.4413	0.9653	0.0949*	
H21	1.2190	0.0950	0.6740	0.0941*	
H22	1.2916	0.0105	0.6502	0.0944*	

### Atomic displacement parameters (Å^2^)

**Table:**

	*U*^11^	*U*^22^	*U*^33^	*U*^12^	*U*^13^	*U*^23^
O1A	0.058 (3)	0.040 (2)	0.063 (3)	−0.006 (2)	−0.024 (2)	−0.005 (2)
C1A	0.055 (4)	0.042 (3)	0.061 (4)	−0.003 (3)	−0.022 (3)	−0.010 (3)
O2A	0.065 (3)	0.045 (3)	0.069 (3)	0.000 (2)	−0.028 (3)	−0.019 (2)
C2A	0.059 (4)	0.055 (4)	0.062 (4)	−0.004 (3)	−0.033 (4)	−0.015 (3)
C3A	0.055 (4)	0.059 (4)	0.059 (4)	−0.011 (3)	−0.022 (3)	−0.013 (3)
C4A	0.044 (3)	0.053 (4)	0.065 (4)	−0.019 (3)	−0.024 (3)	−0.002 (3)
N1A	0.045 (3)	0.043 (3)	0.041 (3)	−0.013 (2)	−0.016 (2)	−0.006 (2)
C5A	0.048 (3)	0.045 (3)	0.038 (3)	−0.018 (3)	−0.017 (3)	−0.004 (2)
C6A	0.048 (3)	0.038 (3)	0.040 (3)	−0.011 (3)	−0.015 (3)	−0.009 (2)
C7A	0.052 (4)	0.042 (3)	0.041 (3)	−0.012 (3)	−0.017 (3)	−0.005 (2)
C8A	0.043 (3)	0.046 (3)	0.041 (3)	−0.010 (3)	−0.018 (3)	−0.003 (3)
C9A	0.052 (4)	0.048 (3)	0.041 (3)	−0.018 (3)	−0.018 (3)	−0.009 (3)
C10A	0.041 (3)	0.044 (3)	0.041 (3)	−0.006 (3)	−0.020 (3)	−0.010 (2)
C11A	0.055 (4)	0.041 (3)	0.050 (4)	−0.017 (3)	−0.022 (3)	−0.001 (3)
C12A	0.051 (4)	0.045 (3)	0.038 (3)	−0.016 (3)	−0.014 (3)	−0.004 (2)
C13A	0.052 (4)	0.048 (4)	0.050 (4)	−0.004 (3)	−0.018 (3)	−0.013 (3)
C14A	0.044 (3)	0.043 (3)	0.048 (3)	−0.006 (3)	−0.016 (3)	−0.011 (3)
C15A	0.048 (3)	0.042 (3)	0.032 (3)	−0.013 (3)	−0.013 (2)	−0.006 (2)
C16A	0.047 (3)	0.046 (3)	0.033 (3)	−0.015 (3)	−0.013 (3)	−0.003 (2)
C17A	0.041 (3)	0.042 (3)	0.035 (3)	−0.009 (2)	−0.014 (2)	−0.007 (2)
C18A	0.042 (3)	0.046 (3)	0.037 (3)	−0.010 (3)	−0.015 (3)	−0.006 (2)
O3A	0.053 (3)	0.040 (2)	0.055 (3)	−0.007 (2)	−0.024 (2)	−0.008 (2)
O4A	0.051 (3)	0.041 (2)	0.044 (2)	−0.016 (2)	−0.017 (2)	−0.0088 (18)
C19A	0.058 (4)	0.038 (3)	0.058 (4)	−0.002 (3)	−0.027 (3)	−0.008 (3)
C20A	0.054 (4)	0.068 (5)	0.046 (4)	−0.016 (3)	−0.016 (3)	−0.019 (3)
C21A	0.067 (5)	0.075 (5)	0.066 (5)	−0.034 (4)	−0.009 (4)	−0.027 (4)
O1B	0.046 (2)	0.033 (2)	0.071 (3)	−0.0049 (19)	−0.026 (2)	−0.011 (2)
C1B	0.046 (4)	0.038 (3)	0.068 (4)	0.000 (3)	−0.021 (3)	−0.011 (3)
O2B	0.056 (3)	0.039 (2)	0.070 (3)	0.001 (2)	−0.026 (3)	−0.020 (2)
C2B	0.045 (3)	0.054 (4)	0.046 (3)	−0.002 (3)	−0.017 (3)	−0.017 (3)
C3B	0.042 (3)	0.050 (4)	0.053 (4)	−0.006 (3)	−0.019 (3)	−0.015 (3)
C4B	0.050 (4)	0.043 (3)	0.058 (4)	−0.004 (3)	−0.030 (3)	−0.013 (3)
N1B	0.035 (2)	0.044 (3)	0.047 (3)	−0.010 (2)	−0.021 (2)	−0.007 (2)
C5B	0.049 (4)	0.041 (3)	0.050 (3)	−0.014 (3)	−0.020 (3)	−0.007 (3)
C6B	0.047 (3)	0.039 (3)	0.048 (3)	−0.011 (3)	−0.017 (3)	−0.008 (3)
C7B	0.042 (3)	0.041 (3)	0.044 (3)	−0.006 (3)	−0.012 (3)	−0.010 (3)
C8B	0.042 (3)	0.042 (3)	0.043 (3)	−0.005 (3)	−0.017 (3)	−0.008 (2)
C9B	0.042 (3)	0.044 (3)	0.042 (3)	−0.012 (3)	−0.020 (3)	−0.008 (2)
C10B	0.040 (3)	0.039 (3)	0.039 (3)	−0.010 (2)	−0.018 (2)	−0.008 (2)
C11B	0.044 (3)	0.034 (3)	0.050 (3)	−0.008 (2)	−0.019 (3)	−0.003 (2)
C12B	0.042 (3)	0.035 (3)	0.049 (3)	−0.012 (2)	−0.015 (3)	−0.005 (2)
C13B	0.049 (4)	0.042 (3)	0.047 (3)	−0.002 (3)	−0.020 (3)	−0.009 (3)
C14B	0.042 (3)	0.051 (4)	0.044 (3)	−0.011 (3)	−0.017 (3)	−0.012 (3)
C15B	0.044 (3)	0.041 (3)	0.040 (3)	−0.012 (3)	−0.016 (3)	−0.007 (2)
C16B	0.033 (3)	0.042 (3)	0.037 (3)	−0.009 (2)	−0.010 (2)	−0.009 (2)
C17B	0.039 (3)	0.042 (3)	0.037 (3)	−0.011 (2)	−0.016 (2)	−0.008 (2)
C18B	0.043 (3)	0.041 (3)	0.041 (3)	−0.010 (3)	−0.017 (3)	−0.006 (2)
O4B	0.054 (3)	0.045 (2)	0.055 (3)	−0.017 (2)	−0.012 (2)	−0.017 (2)
O3B	0.053 (3)	0.036 (2)	0.062 (3)	−0.004 (2)	−0.024 (2)	−0.008 (2)
C19B	0.062 (5)	0.045 (4)	0.071 (5)	0.001 (3)	−0.035 (4)	−0.010 (3)
C20B	0.062 (5)	0.154 (11)	0.070 (6)	−0.041 (6)	−0.002 (4)	−0.068 (7)
C21B	0.062 (5)	0.092 (6)	0.049 (4)	−0.036 (5)	−0.019 (4)	−0.010 (4)
O5	0.060 (3)	0.081 (4)	0.054 (3)	−0.023 (3)	−0.017 (3)	−0.006 (3)
I1A	0.0507 (3)	0.0426 (2)	0.0484 (3)	−0.01080 (19)	−0.0248 (2)	−0.00267 (18)
I1A'	0.0507 (3)	0.0426 (2)	0.0484 (3)	−0.01080 (19)	−0.0248 (2)	−0.00267 (18)
I1B	0.0614 (3)	0.0476 (3)	0.0631 (3)	−0.0168 (2)	−0.0366 (3)	−0.0039 (2)
I1B'	0.0614 (3)	0.0476 (3)	0.0631 (3)	−0.0168 (2)	−0.0366 (3)	−0.0039 (2)

### Geometric parameters (Å, °)

**Table:**

O1A—C1A	1.446 (9)	O1B—C12B	1.358 (7)
O1A—C12A	1.386 (8)	C1B—O2B	1.441 (9)
C1A—O2A	1.450 (9)	C1B—H1B	0.971
C1A—H1A	0.972	C1B—H2B	0.972
C1A—H2A	0.968	O2B—C13B	1.382 (8)
O2A—C13A	1.381 (9)	C2B—C13B	1.374 (10)
C2A—C13A	1.345 (10)	C2B—C14B	1.404 (10)
C2A—C14A	1.419 (10)	C2B—H3B	0.930
C2A—H3A	0.934	C3B—C4B	1.523 (10)
C3A—C4A	1.483 (11)	C3B—C14B	1.511 (9)
C3A—C14A	1.497 (10)	C3B—H4B	0.975
C3A—H4A	0.969	C3B—H5B	0.982
C3A—H5A	0.974	C4B—N1B	1.472 (8)
C4A—N1A	1.485 (9)	C4B—H6B	0.971
C4A—H6A	0.978	C4B—H7B	0.967
C4A—H7A	0.967	N1B—C5B	1.319 (8)
N1A—C5A	1.347 (9)	N1B—C17B	1.398 (7)
N1A—C17A	1.383 (8)	C5B—C15B	1.409 (9)
C5A—C15A	1.396 (9)	C5B—H8B	0.932
C5A—H8A	0.935	C6B—C7B	1.399 (9)
C6A—C7A	1.390 (9)	C6B—C15B	1.398 (9)
C6A—C15A	1.420 (9)	C6B—O4B	1.362 (8)
C6A—O4A	1.370 (7)	C7B—C8B	1.388 (9)
C7A—C8A	1.396 (9)	C7B—O3B	1.359 (8)
C7A—O3A	1.360 (8)	C8B—C9B	1.379 (9)
C8A—C9A	1.376 (10)	C8B—H9B	0.934
C8A—H9A	0.933	C9B—C16B	1.420 (8)
C9A—C16A	1.403 (9)	C9B—H10B	0.934
C9A—H10A	0.933	C10B—C16B	1.406 (8)
C10A—C16A	1.414 (9)	C10B—C17B	1.379 (8)
C10A—C17A	1.371 (8)	C10B—H11B	0.925
C10A—H11A	0.930	C11B—C12B	1.375 (9)
C11A—C12A	1.357 (10)	C11B—C18B	1.419 (8)
C11A—C18A	1.418 (9)	C11B—H12B	0.930
C11A—H12A	0.935	C12B—C13B	1.377 (9)
C12A—C13A	1.383 (10)	C14B—C18B	1.393 (9)
C14A—C18A	1.381 (9)	C15B—C16B	1.410 (8)
C15A—C16A	1.429 (8)	C17B—C18B	1.452 (9)
C17A—C18A	1.486 (9)	O4B—C20B	1.395 (9)
O3A—C19A	1.421 (9)	O3B—C19B	1.439 (9)
O4A—C20A	1.441 (8)	C19B—H13B	0.959
C19A—H13A	0.966	C19B—H14B	0.966
C19A—H14A	0.960	C19B—H15B	0.963
C19A—H15A	0.957	C20B—C21B	1.497 (11)
C20A—C21A	1.481 (10)	C20B—H16B	0.963
C20A—H16A	0.980	C20B—H17B	0.967
C20A—H17A	0.978	C21B—H18B	0.961
C21A—H18A	0.962	C21B—H19B	0.955
C21A—H19A	0.962	C21B—H20B	0.962
C21A—H20A	0.967	O5—H21	0.916
O1B—C1B	1.443 (8)	O5—H22	0.852
			
C1A—O1A—C12A	105.6 (5)	O1B—C1B—O2B	106.1 (5)
O1A—C1A—O2A	105.9 (5)	O1B—C1B—H1B	109.9
O1A—C1A—H1A	110.5	O2B—C1B—H1B	110.5
O2A—C1A—H1A	110.8	O1B—C1B—H2B	109.9
O1A—C1A—H2A	109.7	O2B—C1B—H2B	110.6
O2A—C1A—H2A	109.2	H1B—C1B—H2B	109.7
H1A—C1A—H2A	110.6	C1B—O2B—C13B	104.1 (5)
C1A—O2A—C13A	105.2 (5)	C13B—C2B—C14B	116.8 (6)
C13A—C2A—C14A	117.2 (7)	C13B—C2B—H3B	121.8
C13A—C2A—H3A	121.7	C14B—C2B—H3B	121.4
C14A—C2A—H3A	121.1	C4B—C3B—C14B	111.8 (6)
C4A—C3A—C14A	111.6 (6)	C4B—C3B—H4B	107.8
C4A—C3A—H4A	109.2	C14B—C3B—H4B	109.7
C14A—C3A—H4A	109.2	C4B—C3B—H5B	108.5
C4A—C3A—H5A	107.5	C14B—C3B—H5B	108.9
C14A—C3A—H5A	109.6	H4B—C3B—H5B	110.2
H4A—C3A—H5A	109.8	C3B—C4B—N1B	111.1 (5)
C3A—C4A—N1A	112.0 (6)	C3B—C4B—H6B	108.1
C3A—C4A—H6A	108.0	N1B—C4B—H6B	109.0
N1A—C4A—H6A	110.1	C3B—C4B—H7B	109.5
C3A—C4A—H7A	108.9	N1B—C4B—H7B	109.1
N1A—C4A—H7A	107.2	H6B—C4B—H7B	110.0
H6A—C4A—H7A	110.7	C4B—N1B—C5B	117.3 (5)
C4A—N1A—C5A	117.7 (5)	C4B—N1B—C17B	119.2 (5)
C4A—N1A—C17A	119.8 (6)	C5B—N1B—C17B	123.3 (5)
C5A—N1A—C17A	122.2 (6)	N1B—C5B—C15B	121.7 (6)
N1A—C5A—C15A	121.4 (6)	N1B—C5B—H8B	119.1
N1A—C5A—H8A	119.0	C15B—C5B—H8B	119.2
C15A—C5A—H8A	119.6	C7B—C6B—C15B	118.8 (6)
C7A—C6A—C15A	119.2 (6)	C7B—C6B—O4B	122.5 (6)
C7A—C6A—O4A	122.1 (6)	C15B—C6B—O4B	118.5 (6)
C15A—C6A—O4A	118.7 (6)	C6B—C7B—C8B	119.7 (6)
C6A—C7A—C8A	120.0 (6)	C6B—C7B—O3B	116.6 (6)
C6A—C7A—O3A	116.2 (6)	C8B—C7B—O3B	123.7 (6)
C8A—C7A—O3A	123.8 (6)	C7B—C8B—C9B	122.1 (6)
C7A—C8A—C9A	121.9 (6)	C7B—C8B—H9B	118.2
C7A—C8A—H9A	118.8	C9B—C8B—H9B	119.8
C9A—C8A—H9A	119.3	C8B—C9B—C16B	119.6 (5)
C8A—C9A—C16A	120.0 (6)	C8B—C9B—H10B	120.1
C8A—C9A—H10A	120.7	C16B—C9B—H10B	120.3
C16A—C9A—H10A	119.3	C16B—C10B—C17B	122.9 (5)
C16A—C10A—C17A	122.2 (6)	C16B—C10B—H11B	118.5
C16A—C10A—H11A	118.6	C17B—C10B—H11B	118.6
C17A—C10A—H11A	119.2	C12B—C11B—C18B	117.0 (6)
C12A—C11A—C18A	116.5 (6)	C12B—C11B—H12B	121.4
C12A—C11A—H12A	121.4	C18B—C11B—H12B	121.5
C18A—C11A—H12A	122.1	C11B—C12B—O1B	128.0 (6)
O1A—C12A—C11A	128.2 (6)	C11B—C12B—C13B	121.7 (6)
O1A—C12A—C13A	109.0 (6)	O1B—C12B—C13B	110.1 (6)
C11A—C12A—C13A	122.8 (6)	O2B—C13B—C12B	109.4 (6)
C12A—C13A—O2A	110.2 (6)	O2B—C13B—C2B	127.9 (6)
C12A—C13A—C2A	122.0 (7)	C12B—C13B—C2B	122.6 (6)
O2A—C13A—C2A	127.8 (7)	C3B—C14B—C2B	120.1 (6)
C3A—C14A—C2A	120.7 (6)	C3B—C14B—C18B	118.4 (6)
C3A—C14A—C18A	118.5 (6)	C2B—C14B—C18B	121.3 (6)
C2A—C14A—C18A	120.8 (6)	C5B—C15B—C6B	120.5 (6)
C6A—C15A—C5A	121.7 (6)	C5B—C15B—C16B	117.5 (6)
C6A—C15A—C16A	120.2 (6)	C6B—C15B—C16B	122.0 (6)
C5A—C15A—C16A	118.2 (6)	C9B—C16B—C15B	117.9 (6)
C15A—C16A—C10A	117.8 (6)	C9B—C16B—C10B	123.8 (5)
C15A—C16A—C9A	118.7 (6)	C15B—C16B—C10B	118.3 (5)
C10A—C16A—C9A	123.4 (6)	N1B—C17B—C10B	116.0 (5)
N1A—C17A—C10A	118.1 (6)	N1B—C17B—C18B	119.4 (5)
N1A—C17A—C18A	117.6 (5)	C10B—C17B—C18B	124.6 (5)
C10A—C17A—C18A	124.3 (6)	C17B—C18B—C11B	118.4 (6)
C17A—C18A—C11A	118.5 (6)	C17B—C18B—C14B	121.0 (6)
C17A—C18A—C14A	120.8 (6)	C11B—C18B—C14B	120.5 (6)
C11A—C18A—C14A	120.7 (6)	C6B—O4B—C20B	117.1 (6)
C7A—O3A—C19A	117.2 (5)	C7B—O3B—C19B	116.7 (5)
C6A—O4A—C20A	113.4 (5)	O3B—C19B—H13B	108.7
O3A—C19A—H13A	109.4	O3B—C19B—H14B	109.9
O3A—C19A—H14A	109.1	H13B—C19B—H14B	109.9
H13A—C19A—H14A	110.2	O3B—C19B—H15B	109.9
O3A—C19A—H15A	109.5	H13B—C19B—H15B	109.2
H13A—C19A—H15A	109.3	H14B—C19B—H15B	109.2
H14A—C19A—H15A	109.3	O4B—C20B—C21B	110.0 (7)
O4A—C20A—C21A	108.7 (6)	O4B—C20B—H16B	109.3
O4A—C20A—H16A	107.8	C21B—C20B—H16B	109.6
C21A—C20A—H16A	109.0	O4B—C20B—H17B	109.0
O4A—C20A—H17A	110.6	C21B—C20B—H17B	109.4
C21A—C20A—H17A	110.7	H16B—C20B—H17B	109.5
H16A—C20A—H17A	109.9	C20B—C21B—H18B	109.3
C20A—C21A—H18A	109.8	C20B—C21B—H19B	109.1
C20A—C21A—H19A	109.2	H18B—C21B—H19B	109.7
H18A—C21A—H19A	109.5	C20B—C21B—H20B	109.1
C20A—C21A—H20A	109.4	H18B—C21B—H20B	109.8
H18A—C21A—H20A	109.9	H19B—C21B—H20B	109.7
H19A—C21A—H20A	109.1	H21—O5—H22	90.7
C1B—O1B—C12B	104.4 (5)		

### Hydrogen-bond geometry (Å, °)

**Table:**

*D*—H···*A*	*D*—H	H···*A*	*D*···*A*	*D*—H···*A*
C9A—H10A···O5	0.93	2.47	3.358 (11)	159
C21B—H20B···O1B^i^	0.96	2.51	3.466 (11)	177

Symmetry codes: (i) *x*, *y*+1, *z*.
